# Accelerated Degradation of Poly(lactide acid)/Poly(hydroxybutyrate) (PLA/PHB) Yarns/Fabrics by UV and O_2_ Exposure in South China Seawater

**DOI:** 10.3390/polym14061216

**Published:** 2022-03-17

**Authors:** Qi Bao, Wingho Wong, Shirui Liu, Xiaoming Tao

**Affiliations:** Institute of Textiles and Clothing, The Hong Kong Polytechnic University, Hong Kong 999077, China; pauki.bao@polyu.edu.hk (Q.B.); edwinwwho@gmail.com (W.W.); shirui.liu@polyu.edu.hk (S.L.)

**Keywords:** PLA/PHB, marine degradation, hydrolysis, UV photooxidation

## Abstract

Marine plastic pollution is emerging as a potential hazard to global ecosystems and human health. Micro-fibers derived from synthetic textiles contribute a considerable proportion of plastic debris. Bio-polymers/bio-plastics have been proposed for the application of apparel products, yet their degradability, fate, durability and related environmental parameters are still elusive and need further exploration. Herein, we report the degradation behavior of poly(lactide acid)/poly(hydroxybutyrate) (PLA/PHB) fabrics, made from PLA/PHB multi-filament yarns, in subtropics marine seawater. The degradation experiments were performed under various parallel conditions including static seawater, aerobic seawater in dark box, aerobic seawater under sunlight, static seawater under ultra-violet light and aerobic seawater under ultra-violet light. Continuous mass loss of PLA/PHB fabrics as the immersion time in the seawater increased was confirmed. The hydrolysis rate of PLA/PHB fabrics accelerated in the presence of UV light and dissolved oxygen in the seawater. Moreover, the tensile strength of the PLA/PHB yarns dropped rapidly by 38.54–68.70% in spite of the mass loss percentage being from 9.57% to 14.48% after 2 weeks’ immersion. All the PLA/PHB fabrics after two weeks’ immersion exhibited similar ATR-IR spectra. Therefore, the degradability of PLA/PHB fabrics, in the marine surface water under the synergistic destructive effect of seawater, UV and dissolved oxygen, provides a pathway for more sustainable textile fibers and apparel products.

## 1. Introduction

Synthetic organic plastics have witnessed rapid growth in the consumer marketplace after their initial development between the 1930s and 1940s [1,2]. In 2015, the global production yield of primary plastics was reported to be over 400 million metric tons (Mt/year), quadrupled over 30 years. This number has been expected to be further increased to about 12,000 Mt by 2050 [3]. To date, 59% of primary plastics have been discarded and disposed directly into natural environments, and merely 7.2% of them have been recycled and reused. The vast majority of plastics are derived from fossil hydrocarbons. Their intrinsic inertness and resistance makes them difficult or impossible for nature to assimilate. Thereby, these plastics have accumulated across all major terrestrial and aquatic ecosystems and will remain for an extremely long-lasting time [4]. For instance, the average lasting time of a plastic bottle is 800 years [5,6]. It is estimated that the cumulated plastic waste of primary and secondary (recycled) plastic on the planet amounted to 6300 Mt in the range from 1950 to 2015.

Most of this abandoned plastics waste ends its life in the marine environment via landfills, inland waterways, wastewater outflows and/or transport by wind or tides [7,8,9]. Quantitatively, 4.8 to 12.7 Mt/year of plastic waste entered the ocean from coastal landside in 2010 [10]. This number was predicted to increase by one order of magnitude in 2025, assuming such production and waste management trends continued. Long-term weathering of plastic causes fragmentation into small bits of plastic with diameters < 5 mm (referred to as microplastics) [11,12]. The principal form of microplastics is fiber (84–85%) [13,14]. Once these microplastics are ingested by marine species/seabirds, they are then transported through the food chain/web via the bioaccumulation process (i.e., the accumulation of a substance in an organism’s tissue due to a greater intake rate than excretion or metabolic rate) [15]. This imposes a great threat to marine wildlife, food safety, public health, tourism and the fishing industry. Increasingly, microplastics pollution has emerged as a looming catastrophe because of their persistent hazards for marine ecosystems [16,17].

To address the plastic pollution problem, one effective and economically feasible resolution is to explore bio-plastics as an alternative to the non-degradable plastics [18]. For example, these kinds of bio-plastics include poly(butylene succinate) (PBS), polyhydroxyalkanoate (PHA), poly(ε-caprolactone) (PCL), poly(butylene adipate-co-teraphthalate (PBAT), etc. Among them, poly(lactide acid) (PLA) and other biopolymers from bacterial fermentation, e.g., poly(hydroxybutyrate) (PHB), have attracted tremendous attention and exhibit great potential in both conventional durable goods and disposable products due to their acceptable thermal and mechanical properties [19,20,21].

In addition, the marine degradation behavior of bio-plastics is highly affected by the local environmental parameters. The degradability might change or even be lost once the environment factors or application status change. For example, the lifetime required for complete degradation of a plastics product and the range of uncertainty increases with its physical dimension. Textiles are among the most common items used in in our daily life, including clothing, bags, bed linen, towels, furniture upholstery, etc. Currently, there is still no effective recycling strategy for recovering synthetic fibers. Thus, it is assumed that all disposed textiles at the end of usage are either placed in landfill or incinerated together with all other municipal solid waste. These discarded textiles have become one of the major sources of microplastic fibers accumulated in the oceans. It is of environmental significance to develop bio-plastics textiles to replace the petrochemical ones. In reality, there have been few but controversial studies on the actual degradation behavior of bio-plastics that have been made into thin film samples, although these bio-plastics have been assumed to have 100% biodegradation rate [22]. For example, Tsuji et al. studied the degradation of PCL, PLA and PHB films at 25 °C for predetermined periods in the water of the Pacific Ocean [23]. The degradation rate of PHB was 9%, but the degradation of the PLA films was insignificant after immersion in the seawater for 10 weeks. Moreover, Bagheri et al. also reported that PCL and PLA did not degrade at all, but approximately 8% degradation was observed for PHB in 365 days [24]. However, to our best knowledge, there is no public literature report on the degradation of PLA-based yarns or fabrics on the end products level up to date.

Herein, the natural degradation behavior of poly(lactide acid)/poly(hydroxybutyrate) (PLA/PHB) fabrics, manufactured from novel bio-based and degradable PLA/PHB multi-filament yarns, in the marine water environment was studied in this work for the first time. It was postulated by the authors that PLA/PHB fabrics are degradable in the marine environment, therefore being likely to reduce the future net input of plastics waste into the marine environment after their large-scale application. The degradation behavior of PLA/PHB fabrics in seawater from the Kowloon Bay was evaluated by their mass loss and mechanical properties of PLA/PHB fabrics/yarns. Furthermore, the degradation mechanism was studied by using molecular spectroscopy, thermal analysis and surface morphology of the PLA/PHB fabrics before and after the degradation process immersed in the seawater.

## 2. Experimental

The PLA/PHB multi-filament yarns were obtained from Ningbo Hesu Fibers Co., Ltd. (Ningbo, China). The polymer blend ratio of PLA/PHB is 70/30 for the melt spinning process to produce fully drawn filament yarns (FDY). The linear density of the filament is 75D/48F, where D is Denier (mass density as gram per 9000 m); F is the number of filaments in an FDY yarn. The knitted fabrics were made by using a circular knitting machine (WUXI ERVA Knitted Fashion Co., Ltd. (Wuxi, China)). The fabrics were used for most of assessment tests. A gauge of 28 needles per inch was adopted for producing single jersey knitted fabrics from the PLA/PHB filament yarns. The filament yarns were fed into the knitting machine directly, and the pretension used was 2–3 cN. The surface area of the PLA/PHB fabric was 3554.78 cm^2^/g, which was calculated according to the following equation:

*S* = 2π*r* × (*L* + *r*) (1)

where *S* is the surface area of the PLA/PHB fabric per gram, *r* is the radius of the filament, which was obtained on an optical microscope, and *L* is the length of total filament per gram.

Attenuated total-reflectance Fourier-transform infrared (ATR-IR) spectra were obtained using a ZnSe prism on a Tensor 27 spectrometer (Bruker, Germany). The experimental conditions employed were as follows: 45 angle of incidence of the internal reflection radiation; wavenumber from 4000 cm^−1^ to 650 cm^−1^; and 32 scans at 4 cm^−1^ resolution were obtained and averaged. The non-isothermal crystallization behaviors of PLA/PHB samples were investigated on a PerkinElmer DSC 8000 differential scanning calorimeter (DSC). The prepared sample was sealed in an aluminum pan and placed alongside an empty aluminum reference pan in the DSC furnace. The sample and the reference pan were heated under nitrogen atmosphere at the same rate by a heating element in the furnace. The samples were undergoing a heating process with heating rate of 20 °C min^−^^1^ from 60 to 200 °C using nitrogen gas at 50 mL min^−^^1^. 

The tenacity, elongation, Young’s modulus and extension energy of PLA/PHB multi-filament yarns were obtained by the single-strand method on an Instron 5566 universal testing machine. According to ASTM D2256 / D2256M-10 (2015) standard, the gauge length was fixed to 250 ± 3 mm (10 ± 0.1 in.), and the tensile tests were performed at a constant extension rate V = 125 mm/min under the ambient condition. At least three samples of each yarn were prepared for measuring yarn tensile properties. All the specimen were examined under a pretension of 0.5 ± 0.1 cN/tex. Before measurement, all yarn samples were conditioned in vacuum oven for 24 h at 60 °C. Tenacity at break and percent elongation at break were calculated automatically from tension data.

Accelerated degradation tests of PLA/PHB yarns and fabrics were carried out in separated natural seawater baths (500 mL). The degradation tests were performed under five unique conditions: immersion in (i) static natural seawater, (ii) aerobic natural seawater (the air flow rate was set to be 4.3 ± 1.0 SLPM) under dark box, (iii) aerobic natural seawater under sunlight (Philips Lifemax 18W × 3), (iv) static natural seawater under ultra-violet light (low-pressure mercury-vapor fluorescent lamps, Philips TL-D 18W × 3, 370 nm) in a specific home-made UV chamber and (v) aerobic natural seawater under ultra-violet light in the same UV chamber. All the seawater was renewed every week. 

## 3. Results and Discussion 

**Figure 1:**
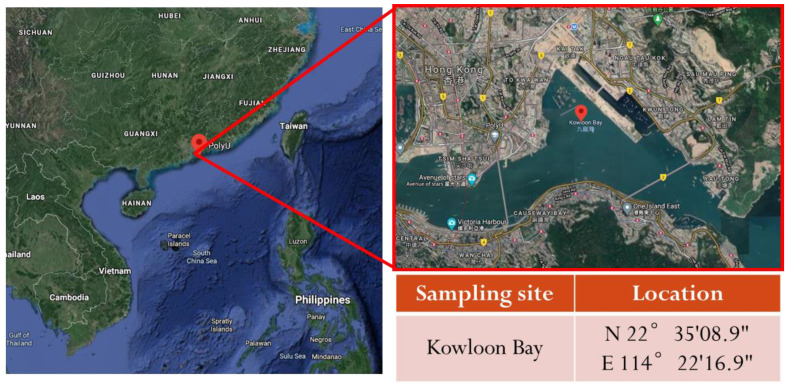
A geographical map showing the seawater sampling site along the coastline and its location.

The experimental seawater was primarily collected from fresh coastal surface seawater of the South China Sea between November and December using a bucket. The seawater sampling site was located in the Kowloon Bay within the Victoria Harbor WCZ (Central Waters), in the middle of the central (VM5) and eastern (VM1) long-term monitoring areas, which were fixed at the east of the Kowloon Peninsula and north of Hong Kong Island, respectively (Figure 1). The seawater was directly used without any further purification. All the physicochemical properties of the seawater were monitored by experienced analysts in a comprehensive marine water quality monitoring system as listed in Table 1. The average water temperature was 24.3 °C, and the average pH was 8.0, slightly alkaline. 

**Figure 2:**
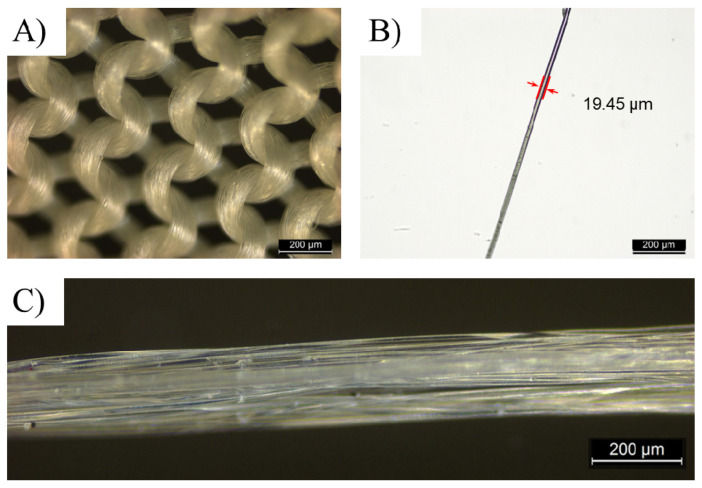
Digital microscopic photograms of (**A**) original PLA/PHB knitted fabric, (**B**) one PLA/PHB fiber from multifilament yarns and (**C**) multi-filament yarns used in fabrics.

Figure 2 shows the apparent geometry of fibers and yarns of the sustainable PLA/PHB fabrics. It clearly shows the integrity of greige-type PLA/PHB fabrics made by the single jersey knitting manufacturing processes, which are light-weight (fabric areal density: 105.17 ± 1.43 g/m²) and commonly applied for producing sportswear and T-shirts in the textile industry. Such a textile fabric has a high surface area in relation to its thickness (0.32 ± 2.76 mm). The PLA/PHB fabrics were made from weaving multi-filament yarns together. The diameter of the yarns was measured in the range from 190 µm to 210 µm. The greater part of fibers displayed a cylindrical structure with an average diameter of 19.45 µm. The yarns were made from multiple long fibers, which were the smallest and fundamental components of this PLA/PHB fabric. These fibers were held together without adhesive to construct yarn in a twistless process, where all the filaments were straight and parallel to each other. An enlarged optical microscope image of the virgin PLA/PHB textile displayed an open knitted structure with loose loops (Figure 2A). The PLA/PHB yarn exhibited a tight structure (Figure 2C). In this case, the PLA/PHB fibers were accessible by sea water in the filament form like most synthetic fibers, which were functionally infinite continuous strands.

A simple and quick method was adopted to determine the degradation percentage and degradation rate of fabrics by measuring the gravimetric mass loss according to the following two equations. The dry mass loss reflected a loss of polymer integrity after the dehydration of the absorbed water. Multiple samples were weighed with a precise five-digit balance and average values were reported here.

(2) $$Mass\;loss\;(\%)=\frac{m_{n}-m_{0}}{m_{0}}\times 100$$

where *m*_n_ is the weight value of the PLA/PHB fabrics after n days immersion in seawater, and *m*_0_ is the initial weight value of the PLA/PHB fabrics before immersion in seawater.

(3) $$Degradation\;rate=\frac{m_{n}-m_{n}-7}{A\times 7}$$

where *m*_n_ is the weight of after n days immersion in seawater, and *A* is the specific surface area of the PLA/PHB fabrics.

**Figure 3:**
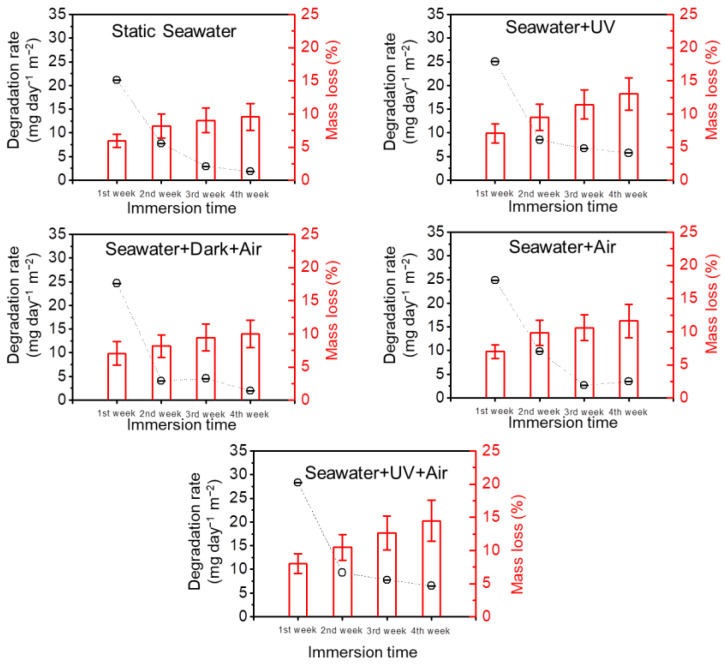
Degradation rate and mass loss as a function of immersion time with various immersion methods.

As shown in Figure 3, all PLA/PHB samples went through an obvious mass loss after immersion, and the degradation rate varied over time. In a typical fulltime experimental period (28 days), the degradation process of all cases could be divided into two stages. In the initial stage (0–14 days), the mass loss rate of the PLA/PHB fabrics was fast. This is because some water-soluble components (~10 wt.%) including some small molecular oligomers, polymer modifiers and/or additives of the PLA/PHB fabrics diffused into the seawater, resulting in such an early-stage reduction of mass. In the following stage (>14 days), the degradation rate of the PLA/PHB fabrics slowed down, and the mass loss was mainly induced by the hydrolysis degradation effect of seawater. The total mass loss of the PLA/PHB fabrics was attributed to the diffusion of soluble molecules and the hydrolysis of seawater. This comparative experiment reveals that the degradation rates of different conditions follow the order: case (UV + Air) > case (UV) > case (Air) > case (Dark + Air) > case (Static seawater). The degradation rates of all the five samples fall in the range from 4.0 mg day^−1^ m^−2^ to 28.5 mg day^−1^ m^−2^. The UV + Air case almost maintained the highest degradation rate, and 14.48% mass loss of the PLA/PHB fabric was achieved within 28 days under ambient conditions in the presence of both UV and DO. In contrast, the mass loss of the PLA/PHB fabric in the static seawater without extra UV and DO was 9.57 wt.%, and the according degradation rate was lowest among the five cases. By comparison, this result indicates that the presence of either UV light or dissolved oxygen (DO) could accelerate such a degradation reaction of PLA/PHB fabrics. Both UV light and dissolved oxygen (DO) play a vital role in the accelerated degradation reaction of the PLA/PHB fabrics. Moreover, there is a synergistic interaction between UV and DO. This is because the mass loss percentage of the UV + Air case was much larger than that of either the UV case or the Air case.

**Figure 4:**
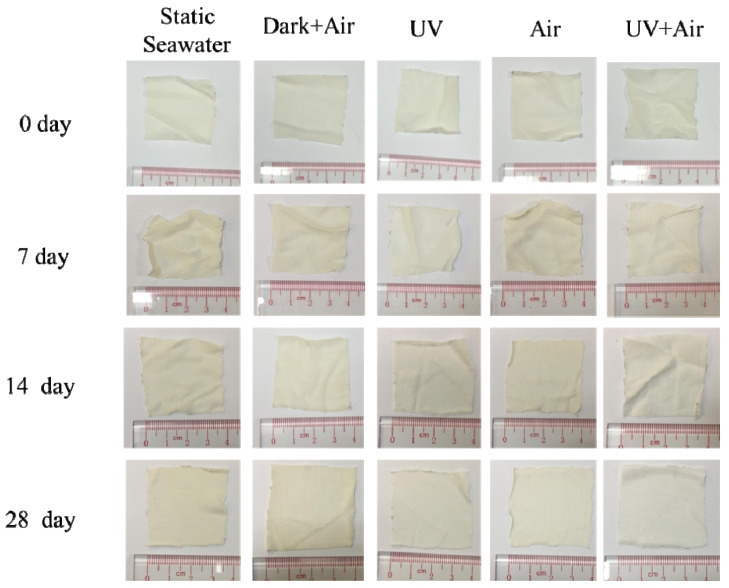
Visual images of multi-filament PLA/PHB fabrics before (0 days) and after 7 days, 14 days and 28 days degradation and immersion time under different conditions. Each sample was dried at 60 °C in vacuum for 48 h.

Figure 4 shows the optical photographs of the PLA/PHB fabrics before and after the immersion in seawater under different conditions for 1–4 weeks. All the samples kept their structural integrity even after immersion for 4 weeks. No obvious hole or crack has been identified by visual observation, and the structural integrity at the fabric level has been maintained. There is no significant change in the apparel color of the PLA/PHB fabrics. However, the fabrics after exposure to UV radiations appear much whiter after immersion as a result of the UV bleaching effect. This result shows good agreement with Yu et al.’s report on the degradation behavior of the transparent PLA films [25]. Such an appearance change might be induced by the variety of the internal crystal structure (e.g., crystallinity) of PLA/PHB and/or the change of chemical bond (e.g., the so-called “five-fingered” character in ester compounds [26]), since PLA exhibits slight to broad medium UV absorption bands below 400 nm through n-π* transition of carboxyl groups [27,28].

**Figure 5:**
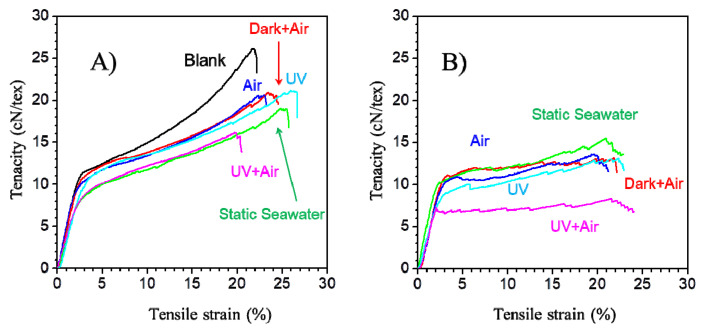
Evolution of tensile behavior of multi-filament PLA/PHB yarns (**A**) before (blank) and after 7 days immersed in natural seawater under different conditions; (**B**) after 14 days immersed in natural seawater under various conditions.

The understanding of how different marine environmental aging factors affect the mechanical behavior of PLA/PHB fabrics remains highly fragmented and incoherent. As shown in Figure 5, the typical tactile curves of the PLA/PHB yarn under continuously tensile load show that the applied force initially increases rapidly and linearly up to a certain extension, after which further extension requires smaller increases in the applied force. Thus, this tensile curve can be divided into two distinct zones: (i) the linear zone (elastic area) and (ii) the postelastic zone (plastic area) [29]. The experimental results have also been derived from the experimental curves and summarized in Table 2. After one week’s immersion, all the PLA/PHB yarns showed similar rigidity as well as yield strength, and no significant variation was found in the linear zone. The Young’s modulus was calculated as a slope of such a linear portion of experimental curve, whose value remained in the range from 455.97 ± 79.53 cN/tex to 523.17 ± 31.46 cN/tex. It is obvious that the cohesion between fibers without twistiness was low, in that the friction of the fibers was negligible. However, the degradation had significant influence on their plastic behavior, and the tenacity decreased drastically from 2.62 ± 0.02 cN/dtex to 1.56 ± 0.15 cN/dtex in the UV + Air case. After 2 weeks’ immersion, such a tendency went on continuously, and the PLA/PHB yarns became much softer. The force–deformation curve remained steady in the nonlinear zone, and the PLA/PHB yarns kept a constant stress strength value until break at higher strains, which also meant that there was a critical breaking strength value during the stretching process. The tensile strength of PLA/PHB yarns dropped by 38.5% (Static seawater), 46.2% (Dark + Air), 42.7% (Air), 45.8% (UV) and 68.7% (UV + Air), respectively. After 3 weeks or more, the PLA/PHB yarns lost their plastic behavior and exhibited a brittle fracture with a low extension (<10%) due to lack of energy-absorbing characteristics leading to catastrophic fracture. Generally, the non-crystalline, highly deformable amorphous phase of PLA/PHB blends affects their ductility [30]. Thus, the hydrolysis degradation predominantly deteriorated the amorphous phase of PLA/PHB blends, making them fragile and susceptible to rupture. Olga Mysiukiewicz et al. evidenced that water penetration was higher in PLA composites and hydrolytic degradation occurred not only in the surface but also throughout the entire body [31]. However, the higher crystalline region, coupled with lower mobility of long polymer chains, hindered water diffusing into the gaps between polymer chains [32]. Thus, the Young’s modulus values have been maintained after aging.

**Figure 6:**
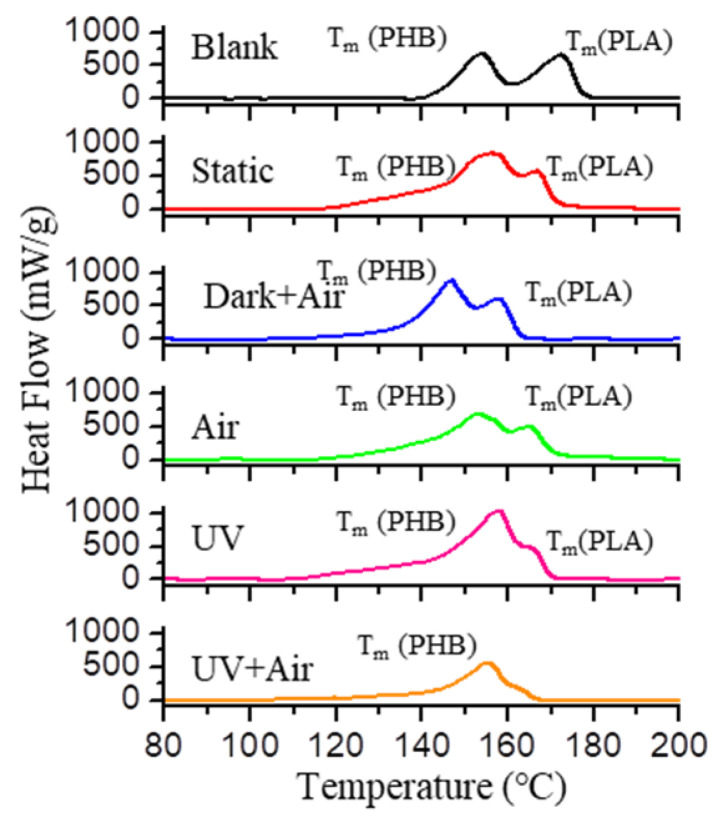
DSC thermograms of PLA/PHB fabrics at a heating rate of 20 °C/min before and after different conditions.

Meanwhile, the tensile strength at break of the PLA/PHB yarns decreased from 2.62 ± 0.02 cN/detex to 0.82 ± 0.03 cN/detex after 2 weeks’ exposure in the presence of UV and O_2_. Generally, such decrement in the tensile strength at break and tensile strength at yield was correlated to the decrease in the crystallinity of polymers and their blends. This was also indicated by the change of apparel color, as mentioned previously. Thereafter, DSC was carried out to measure the variety of crystallinity of PLA/PHB fabrics after aging process, as shown in Figure 6. There were two distinct endothermic peaks observed in the heating process from 60 °C to 200 °C. The lower one of them was attributed to the melting temperature range of PHB (*T*_m_ PHB), and the higher one was the melting temperature range of PLA (*T*_m_ PLA). By comparison, the size of the endothermic peak of PHB was maintained after aging, whereas the size of the endothermic peak of PLA decreased gradually to almost zero. The integrated area of the endothermic peak in the DSC curves was applied to calculate the melting enthalpy of polymers (Δ*H*_m_). The degree of crystallinity (*X*_c_) was calculated using the following equation:

(4) $$Xc=\frac{{\Delta H}_{m}}{{\Delta H}_{m}^{0}}$$

where Δ*H*^0^_m_ represents theorical melting enthalpy of 100% crystalline PLA and PHB, which are 93 J/g and 146 J/g, respectively. The crystallization degree (*X*_c_) values of PHB and PLA before aging were calculated to be 15% and 12%, respectively. Obviously, there was significant reduction in the crystallization degree of PLA after aging process. However, there are two possible reasons to explain this phenomenon; that is, the degree of crystallinity of PLA component decreased, and/or the weight content of PLA in this binary blend decreased.

**Figure 7:**
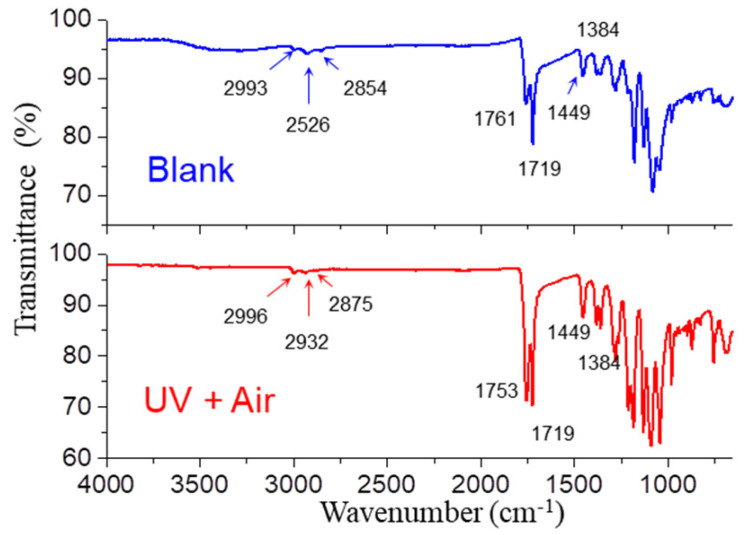
FTIR-ATR spectra of PLA/PHB textiles before and after 14 days immersion in natural seawater under both UV light radiation and air gas bubbling condition.

The PLA/PHB multifilament fabrics after two weeks’ aging were also characterized and compared by non-destructive ATR-IR analysis, as shown in Figure 7. In the ATR-IR spectra of PLA/PHB, the typical stretching vibration peak of O–H of hydroxyl groups was evident at 3287 cm^−1^. The (anti)symmetric C–H of primary (–CH_3_), secondary (–CH_2_–) and tertiary alkyl groups were observed in the range from 3033 cm^−1^ to 2812 cm^−1^. The symmetric stretching (ν_s_) and antisymmetric stretching (ν_as_) vibrations of C=O (–COOR) were observed at 1761/1753 cm^−1^ and 1719 cm^−1^, respectively. The most distinguishing variation after aging process occurred in the carbonyl bonds of the ester bonds, which were most susceptible to hydrolysis. This is because the hydrolysis reactions of ester bonds in the aqueous solution were usually consistent with mechanisms involving cooperative catalysis by autoionization-generated hydroxide and hydronium, a process known to have an activation free energy of 23.8 kcal/mol; thus, the total activation barriers were reduced, and the rate of the ester hydrolysis process was accelerated, as revealed by molecular dynamics simulations [33]. The variation of two absorption peaks located within the region 1600–1800 cm^−1^ might be induced by carboxylate–metal ion interactions (–COOMe) after the formation of an inner-sphere complex [34]. The slight polarity made PLA much more vulnerable to both hydrolysis and photolysis degradation compared with PHB. Generally, an increase in polymerization degree would induce an increase in the peak density of ν_as_ as well as a slight peak shift of ν_s_ from a higher wavenumber (1761 cm^−1^) to a lower one (1753 cm^−1^). It is impossible that the polymerization degree of PLA/PHB has been increased in such conditions. However, it is rational that some PLA/PHB polymers with low polymerization degree dissolved into seawater. This is also supported by the fact that the broad and slight adsorption of –OH groups ascribed to the end groups of PLA/PHB polymers located in the range of 3683–3033 cm^−1^ almost disappeared after aging process. Three peaks located at 1449 cm^−1^, 1384 cm^−1^ and 1357 cm^−1^ were ascribed to the C–H bends of saturated alkyl groups such as –CH_3_ and –CH_2_–. Typical C–O and O–C(O)–C stretching vibrations at 1278 cm^−1^ and 1081 cm^−1^ were also found in the ATR-IR spectra of PLA/PHB blend. All the aged PLA/PHB multifilament fabrics after two weeks or more exhibited similar ATR-IR spectra of PLA/PHB fabrics under both UV and air exposure. 

**Figure 8:**
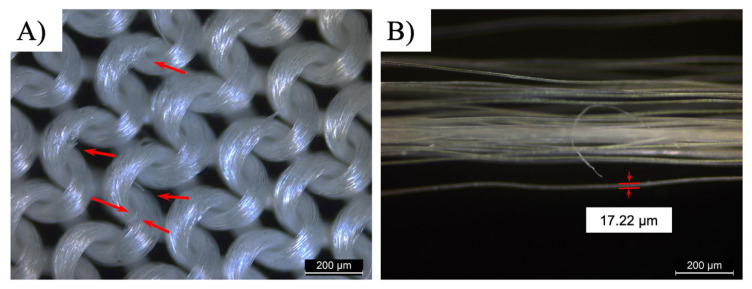
Digital microscopic images of (**A**) PLA/PHB woven fabrics, (**B**) multi-filament yarns used in fabrics after 21 days immersion in seawater under UV + Air condition.

As the microscopic observation shows in Figure 8, severe damages were found in the PLA/PHB multifilament fabrics after aging process, although the physical appearance still looked intact. Some continuous PLA/PHB fibers in the warp yarns broke off entirely in the stress concentrated area of the multifilament fabrics, in particular the max convex part of the knitted structure. Persistent UV exposure and dissolved oxygen attacks produced extensive damages under the photolysis and hydrolysis effects. The neighboring fiber in the fiber bundle became estranged due to the lack of effective inter-fiber bonding, making the PLA/PHB yarns collapse. Meanwhile, the average diameter of each fiber shrunk to be 17.22 ± 1.57 µm from the original 19.45 ± 0.06 µm. 

**Figure 9:**
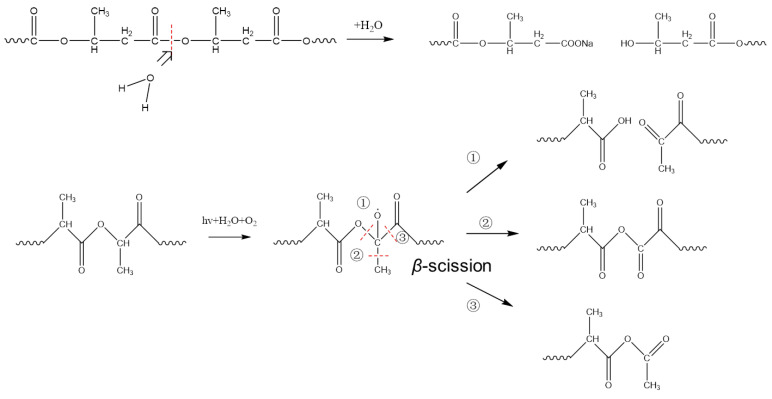
Degradation mechanism of PLA/PHB fabrics after immersion in natural seawater.

As shown in Figure 9, alkaline hydrolysis in aqueous solution mainly leads to chain scission in the C–O bonds of the ester structure of PLA and/or PHB via a two-step B_AC_2 mode, which leads to a decrease in molecular weight and mechanical properties [35,36]. Ikada et al. previously reported UV irradiation deteriorated the structure of PLA via a “Norrish type II” photodegradation mechanism, which formed more carboxylic acid and a new group of C=C [37,38]. However, there is no distinct new absorbance of C=C vibration at ≈1600 cm^−1^ in this work. On the other hand, PLA was susceptible to UV photodegradation, making the degradation of PLA polymer accelerated. Thereafter, two photo-degradation mechanisms have been proposed to explain the anaerobic photodegradation of PLA [39,40]. Firstly, as the PLA-based materials were exposed to UV-light in the range 300–400 nm, the tertiary hydrogen was abstracted from the PLA chain. The tertiary carbon radicals were formed in the *α*-position of the ester bond, and then reacted with oxygen to generate macroradicals or macroradical alkoxy after a series of photolysis processes. These intermediate macroradicals were unstable to undergo the so-called “*beta*-scission” to decompose into three different fragments. The third reaction route dominated the *beta*-scission reaction, which induced the formation of anhydride-groups-based fragments. This could give a reasonable explanation of the ATR-IR results as well as the formation of intermediate radicals during the degradation process, as revealed by EPR imaging [40].

## 4. Conclusions

In this work, the degradability of PLA/PHB yarns/fabrics after immersion in South China Seawater was confirmed by mass loss of dried PLA/PHB fabrics. The accelerated degradation of PLA/PHB yarns/fabrics by extra artificial UV and O_2_ exposure has also been performed in parallel aqueous semi-laboratory environments to simulate harsh marine surface environment (Static seawater, Seawater + UV, Seawater + Dark + Air, Seawater + Air, and Seawater + UV + Air). Two key physicochemical parameters (UV and O_2_) promoting the degradation process have been identified, since the degradation rate of different conditions follows the order: (UV + Air) > (UV) > (Air) > (Dark + Air) > (Static seawater). Assuming the degradation rate was fixed at the 4th weeks’ value, the total degradation time (100% degradation) was calculated to be 374 days (UV + Air), 432 days (UV), 686 days (Air), 1250 days (Dark + Air) and 1273 days (Static seawater), respectively. There is a synergistic interaction between UV and dissolved O_2_, contributing to such a UV-enhanced oxidation process. Such a degradation rate of PLA/PHB fabrics was almost 100 times faster than that of some non-degradable plastics products. Although the mass loss was merely less than 10% within the initial 2 weeks, the decay of mechanical property was significant, particularly in the plastic deformation. The tensile strength was reduced by 38.5–68.7% after 2 weeks’ immersion, whereas the Young’s modules were reduced by no more than 23.2%. Such a mechanical degradation of PLA/PHB fabrics was closely correlated to the change of crystallinity of PLA component and was detrimental to assure in-service resistance as an engineering material. The degradation of PLA/PHB fabrics mainly follows the UV enhanced hydrolytic pathway by the gradual scission of the ester bonds of PLA/PHB materials, as evidenced by ATR-IR results.

## Figures and Tables

**Table 1:** Some basic physicochemical properties of seawater sample.

Hydrochemical Parameters	Average Value (Range)	Unit
Temperature	24.3 (19.2–29.2)	°C
Salinity (psu)	32.1 (30.1–33.3)	‰
Dissolved Oxygen (DO)	5.8 (4.4–7.3)	mg/L
pH	8.0 (7.6–8.2)	
5-day Biochemical Oxygen Demand (BOD_5_)	0.6 (0.2–2.0)	
*Escherichia coli*	240 (56–470)	CFU/100 mL

**Table 2:** Tensile properties of multi-filament PLA/PHB yarns after immersion in seawater under different conditions.

Duration (Day)	Condition	Young’s Modulus (cN/tex)	Tenacity at Max Load (cN/dtex)	Max Elongation (%)	Energy at Max Load (J)
0	Blank	510.09 ± 19.36	2.62 ± 0.02	23.14 ± 1.34	0.13 ± 0.01
7	Static	515.13 ± 47.78	2.24 ± 0.03	25.52 ± 1.66	0.12 ± 0.01
	Dark + Air	523.17 ± 31.46	2.10 ± 0.02	23.90 ± 0.54	0.11 ± 0.00
	Air	455.97 ± 79.53	1.78 ± 0.24	23.49 ± 1.01	0.10 ± 0.01
	UV	475.99 ± 38.37	1.98 ± 0.14	24.58 ± 1.73	0.11 ± 0.01
	UV + Air	512.30±56.66	1.56 ± 0.15	21.49 ± 1.05	0.08 ± 0.01
14	Static	529.35 ± 13.99	1.61 ± 0.10	23.03 ± 1.98	0.10 ± 0.01
	Dark + Air	391.49 ± 175.76	1.41 ± 0.14	22.50 ± 0.94	0.08 ± 0.01
	Air	479.30 ± 25.73	1.50 ± 0.24	19.56 ± 4.70	0.08 ± 0.02
	UV	420.03 ± 135.28	1.42 ± 0.20	21.50 ± 2.23	0.08 ± 0.02
	UV + Air	485.18±53.06	0.82 ± 0.03	14.03 ± 9.94	0.03 ± 0.02

## Data Availability

Not applicable.

## References

[1] Galloway T.S., Cole M., Lewis C. (2017). Interactions of microplastic debris throughout the marine ecosystem. Nat. Ecol. Evol..

[2] Thompson R.C., Olsen Y., Mitchell R.P., Davis A., Rowland S.J., John A.W.G., McGonigle D., Russell A.E. (2004). Lost at Sea: Where Is All the Plastic?. Science.

[3] Geyer R., Jambeck J.R., Law K.L. (2017). Production, use, and fate of all plastics ever made. Sci. Adv..

[4] Bergmann M., Tekman M.B., Gutow L. (2017). Sea change for plastic pollution. Nature.

[5] Ward C.P., Reddy C.M. (2020). Opinion: We need better data about the environmental persistence of plastic goods. Proc. Natl. Acad. Sci. USA.

[6] Chamas A., Moon H., Zheng J., Qiu Y., Tabassum T., Jang J.H., Abu-Omar M., Scott S.L., Suh S. (2020). Degradation Rates of Plastics in the Environment. ACS Sustain. Chem. Eng..

[7] Jambeck J.R., Geyer R., Wilcox C., Siegler T.R., Perryman M., Andrady A., Narayan R., Law K.L. (2015). Plastic waste inputs from land into the ocean. Science.

[8] Bergmann M., Mützel S., Primpke S., Tekman M.B., Trachsel J., Gerdts G. (2019). White and wonderful? Microplastics prevail in snow from the Alps to the Arctic. Sci. Adv..

[9] Rochman C.M., Hoellein T. (2020). The global odyssey of plastic pollution. Science.

[10] Lau W.W.Y., Shiran Y., Bailey R.M., Cook E., Stuchtey M.R., Koskella J., Velis C.A., Godfrey L., Boucher J., Murphy M.B. (2020). Evaluating scenarios toward zero plastic pollution. Science.

[11] Andrady A.L. (2011). Microplastics in the marine environment. Mar. Pollut. Bull..

[12] Martin C., Baalkhuyur F., Valluzzi L., Saderne V., Cusack M., Almahasheer H., Krishnakumar P.K., Rabaoui L., Qurban M.A., Arias-Ortiz A. (2020). Exponential increase of plastic burial in mangrove sediments as a major plastic sink. Sci. Adv..

[13] Suaria G., Achtypi A., Perold V., Lee J.R., Pierucci A., Bornman T.G., Aliani S., Ryan P.G. (2020). Microfibers in oceanic surface waters: A global characterization. Sci. Adv..

[14] Salvador Cesa F., Turra A., Baruque-Ramos J. (2017). Synthetic fibers as microplastics in the marine environment: A review from textile perspective with a focus on domestic washings. Sci. Total Environ..

[15] Mishra S., Rath C.c., Das A.P. (2019). Marine microfiber pollution: A review on present status and future challenges. Mar. Pollut. Bull..

[16] Gago J., Carretero O., Filgueiras A.V., Viñas L. (2018). Synthetic microfibers in the marine environment: A review on their occurrence in seawater and sediments. Mar. Pollut. Bull..

[17] Ju S., Shin G., Lee M., Koo J.M., Jeon H., Ok Y.S., Hwang D.S., Hwang S.Y., Oh D.X., Park J. (2021). Biodegradable chito-beads replacing non-biodegradable microplastics for cosmetics. Green Chem..

[18] Narancic T., O’Connor K.E. (2017). Microbial biotechnology addressing the plastic waste disaster. Microb. Biotechnol..

[19] Aydemir D., Gardner D.J. (2020). Biopolymer blends of polyhydroxybutyrate and polylactic acid reinforced with cellulose nanofibrils. Carbohydr. Polym..

[20] Arrieta M.P., Perdiguero M., Fiori S., Kenny J.M., Peponi L. (2020). Biodegradable electrospun PLA-PHB fibers plasticized with oligomeric lactic acid. Polym. Degrad. Stab..

[21] Huang X., Tao X., Zhang Z., Chen P. (2017). Properties and performances of fabrics made from bio-based and degradable polylactide acid/poly (hydroxybutyrate- co-hydroxyvalerate) (PLA/PHBV) filament yarns. Text. Res. J..

[22] Wang G.-X., Huang D., Ji J.-H., Völker C., Wurm F.R. (2021). Seawater-Degradable Polymers—Fighting the Marine Plastic Pollution. Adv. Sci..

[23] Tsuji H., Suzuyoshi K. (2002). Environmental degradation of biodegradable polyesters 2. Poly(epsilon-caprolactone), poly (R)-3-hydroxybutyrate, and poly(L-lactide) films in natural dynamic seawater. Polym. Degrad. Stabil..

[24] Bagheri A.R., Laforsch C., Greiner A., Agarwal S. (2017). Fate of So-Called Biodegradable Polymers in Seawater and Freshwater. Glob. Chall..

[25] Wang Y.Y., Yu H.-Y., Yang L., Abdalkarim S.Y.H., Chen W.-L. (2019). Enhancing long-term biodegradability and UV-shielding performances of transparent polylactic acid nanocomposite films by adding cellulose nanocrystal-zinc oxide hybrids. Int. J. Biol. Macromol..

[26] Tertyshnaya Y.V., Podzorova M.V. (2020). Effect of UV Irradiation on the Structural and Dynamic Characteristics of Polylactide and Its Blends with Polyethylene. Russ. J. Phys. Chem. B.

[27] Can U., Kaynak C. (2020). Performance of polylactide against UV irradiation: Synergism of an organic UV absorber with micron and nano-sized TiO_2_. J. Compos. Mater..

[28] González-López M.E., Martín del Campo A.S., Robledo-Ortíz J.R., Arellano M., Pérez-Fonseca A.A. (2020). Accelerated weathering of poly(lactic acid) and its biocomposites: A review. Polym. Degrad. Stab..

[29] Lawrence C., Sinclair R. (2015). Chapter 10—Fibre to Yarn: Filament Yarn Spinning. Textiles and Fashion.

[30] Datta D., Halder G. (2019). Effect of media on degradability, physico-mechanical and optical properties of synthesized polyolefinic and PLA film in comparison with casted potato/corn starch biofilm. Process Saf. Environ. Prot..

[31] Mysiukiewicz O., Barczewski M., Skórczewska K., Szulc J., Kloziński A. (2019). Accelerated Weathering of Polylactide-Based Composites Filled with Linseed Cake: The Influence of Time and Oil Content within the Filler. Polymers.

[32] Zhou Q., Xanthos M. (2008). Nanoclay and crystallinity effects on the hydrolytic degradation of polylactides. Polym. Degrad. Stab..

[33] Gunaydin H., Houk K.N. (2008). Molecular Dynamics Prediction of the Mechanism of Ester Hydrolysis in Water. J. Am. Chem. Soc..

[34] Bao Q., Zhang D., Lv D., Wang P. (2012). Effects of two main metabolites of sulphate-reducing bacteria on the corrosion of Q235 steels in 3.5 wt.% NaCl media. Corros. Sci..

[35] Zhan C.-G., Landry D.W., Ornstein R.L. (2000). Energy Barriers for Alkaline Hydrolysis of Carboxylic Acid Esters in Aqueous Solution by Reaction Field Calculations. J. Phys. Chem. A.

[36] Zhan C.-G., Landry D.W., Ornstein R.L. (2000). Reaction Pathways and Energy Barriers for Alkaline Hydrolysis of Carboxylic Acid Esters in Water Studied by a Hybrid Supermolecule-Polarizable Continuum Approach. J. Am. Chem. Soc..

[37] Bocchini S., Fukushima K., Blasio A.D., Fina A., Frache A., Geobaldo F. (2010). Polylactic Acid and Polylactic Acid-Based Nanocomposite Photooxidation. Biomacromolecules.

[38] Ikada E. (1997). Photo- and Bio-degradable Polyesters. Photodegradation Behaviors of Aliphatic Polyesters. J. Photopolym. Sci. Technol..

[39] Gardette M., Thérias S., Gardette J.-L., Murariu M., Dubois P. (2011). Photooxidation of polylactide/calcium sulphate composites. Polym. Degrad. Stab..

[40] Lesaffre N., Bellayer S., Vezin H., Fontaine G., Jimenez M., Bourbigot S. (2017). Recent advances on the ageing of flame retarded PLA: Effect of UV-light and/or relative humidity. Polym. Degrad. Stab..

